# Oral Delivery of Probiotics Expressing Dendritic Cell-Targeting Peptide Fused with Porcine Epidemic Diarrhea Virus COE Antigen: A Promising Vaccine Strategy against PEDV

**DOI:** 10.3390/v9110312

**Published:** 2017-10-25

**Authors:** Xiaona Wang, Li Wang, Xuewei Huang, Sunting Ma, Meiling Yu, Wen Shi, Xinyuan Qiao, Lijie Tang, Yigang Xu, Yijing Li

**Affiliations:** 1 College of Veterinary Medicine, Northeast Agricultural University, Harbin 150030, China; xiaonawang0319@163.com (X.W.); wanglicau@163.com (L.W.); huangxuewei126@126.com (X.H.); masunting@163.com (S.M.); yu19890130@126.com (M.Y.); qiaoxinyuan@126.com (X.Q.); tanglijie@neau.edu.cn (L.T.); 2 College of Animal Science and Technology, Northeast Agricultural University, Harbin 150030, China; wenshi_china@163.com; 3 Heilongjiang Key Laboratory for Animal Disease Control and Pharmaceutical Development, Harbin 150030, China

**Keywords:** *Lactobacillus*, PEDV COE antigen, dendritic cell-targeting peptide, oral vaccine

## Abstract

Porcine epidemic diarrhea virus (PEDV), an enteric coronavirus, is the causative agent of porcine epidemic diarrhea (PED) that damages intestinal epithelial cells and results in severe diarrhea and dehydration in neonatal suckling pigs with up to 100% mortality. The oral vaccine route is reported as a promising approach for inducing protective immunity against PEDV invasion. Furthermore, dendritic cells (DCs), professional antigen-presenting cells, link humoral and cellular immune responses for homeostasis of the intestinal immune environment. In this study, in order to explore an efficient oral vaccine against PEDV infection, a mucosal DC-targeting oral vaccine was developed using *Lactobacillus casei* to deliver the DC-targeting peptide (DCpep) fused with the PEDV core neutralizing epitope (COE) antigen. This probiotic vaccine could efficiently elicit secretory immunoglobulin A (SIgA)-based mucosal and immunoglobulin G (IgG)-based humoral immune responses via oral vaccination in vivo. Significant differences (*p* < 0.05) in the immune response levels were observed between probiotics expressing the COE-DCpep fusion protein and COE antigen alone, suggesting better immune efficiency of the probiotics vaccine expressing the DC-targeting peptide fused with PEDV COE antigen. This mucosal DC-targeting oral vaccine delivery effectively enhances vaccine antigen delivery efficiency, providing a useful strategy to induce efficient immune responses against PEDV infection.

## 1. Introduction

Porcine epidemic diarrhea virus (PEDV) infection, causing an acute and highly contagious enteric disease in nursing pigs with up to 100% mortality, mainly results in intestinal-epithelial-cell damage [1]. Currently, an RNA vaccine and an inactivated whole-virus vaccine of porcine epidemic diarrhea were developed at Harrisvaccines^TM^ and Zoetis (Florham Park, NJ, USA), respectively, widely used and considered to be effective [2]. PEDV mainly gains entry to the host via the intestinemucosal surfaces of the piglets. Therefore, it is important to develop oral mucosa vaccines that can elicit effective mucosal immune responses against PEDV infection. Nevertheless, to reach the mucosal immunity-related sites in sufficient amounts, oral mucosal vaccines need to withstand harsh digestive environments, which are proving to be a big challenge for mucosal vaccine development [3]; therefore, effective antigen delivery vehicles and the adjuvant limit the availability of oral mucosal vaccines for stimulation of immunity [4].

Currently, there is increasing interest in the development of lactic acid bacteria (LAB)-based oral vaccines against enteric viruses; this approach is crucial for the effective induction of a mucosal immune response. *Lactobacillus* strains have many characteristics that make them promising candidates as delivery systems for presenting compounds with antigens of interest to the mucosa, in particular vaccines [5]; for example, *Lactobacilli* can survive in (and colonize) the upper gastrointestinal tract and exert an intrinsic adjuvant activity [6,7]. Moreover, recent reports have shown that *Lactobacillus* species can effectively elicit production of secretory immunoglobulin A (SIgA), induce anti-inflammatory responses, activate innate cells, and regulate the balance between T cell subset responses [8,9]. On the other hand, in order to enhance the deliver efficiency of vaccine antigens to mucosal immune tissues via oral administration, a dendritic cell (DC)-targeting mucosal vaccine was suggested as a realistic approach for oral vaccination to induce high mucosal immune responses against infection [10]. DCs, widely distributed beneath the gastrointestinal epithelium, are professional antigen-presenting cells serving as a portal for virus invasion [11]; therefore, DCs are an early target for virus attachment. Intestinal DC subsets regulate of the intestinalimmune homeostasis through linking humoral and cellular immune responses [12]. It was confirmed that an intestinal DC-targeting oral vaccine could elicit highly efficient antigen-specific immune responses, protecting the mucosal membrane against pathogen infection [13]. Currently, the vaccine strategy of using *Lactobacillus* to express DC-targeting peptide (DCpep) conjugated with *Bacillus anthracis* PA antigen [14] and Newcastle disease virus HN antigen has been investigated to show improved immunogenicity.

Moreover, secretory IgA (SIgA) is the predominant antibody isotype on all mucosal surfaces and prevents bacteria and viruses from infecting the intestinal mucosal barrier [15]. SIgA establishes the first line of defense on the mucosal surface to block adhesion and invasion of infectious agents [16]. Because the mucosal surface is the initial infection site for PEDV, especially in the intestinal mucosa, it would be interesting to develop oral mucosal vaccines that elicit an effective mucosal immune response against PEDV infection. The Spike (S) glycoprotein of PEDV that mediates receptor binding and membrane fusion [17] harbors several neutralizing epitopes [18], particularly the core neutralizing epitope (COE), which can induce neutralizing antibodies against PEDV [19]. The COE has been successfully expressed for vaccine purposes in plants [20] and in lactic acid bacteria [21].

In the present study, a recombinant *Lactobacillus casei* expressing the DC-targeting peptide conjugated with PEDV COE antigen was developed, and its immunogenicity upon administration as an oral vaccine was evaluated.

## 2. Materials and Methods

### 2.1. Bacterial Strain, Virus, and Plasmid

**Figure 1:**
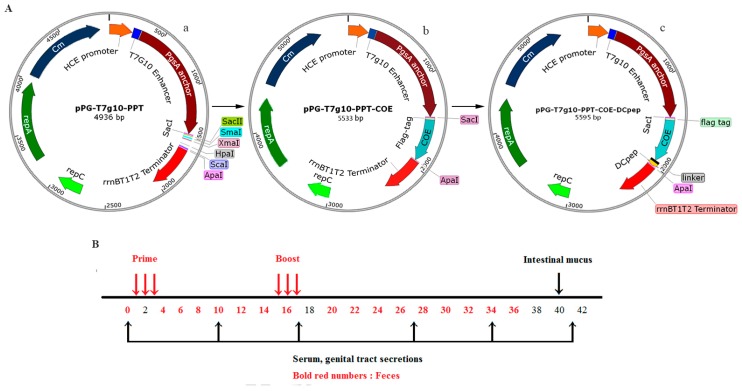
(**A**) Schematic diagram of the construction of recombinant plasmids. (**a**) The constitutive cell surface expression plasmid pPG-T7g10-PPT, (**b**) recombinant plasmid pPG-T7g10-PPT-COE containing core neutralizing epitope (COE), (**c**) recombinant plasmid pPG-T7g10-PPT-COE-DCpep containing the fusion gene *COE-DC*-targeting peptide (DCpep); (**B**) Details of the immunization schedule for recombinant lactic acid bacteria and sampling post-immunization. Red arrows indicate the time points of primary immunization and booster immunization; black arrow (above the line) indicate the time point of intestinal mucus collection, and black arrows (below the line) indicate the time points of serum and genital tract secretions collection.

*Lactobacillus casei* ATCC 393 (*L. casei* 393) was cultured in our laboratory in de Man, Rogosa and Sharp (MRS) broth at 37 °C without shaking. PEDV LJB/15 strain was isolated and identified from clinical samples by our laboratory and was propagated in Vero cells at 37 °C with 5% CO_2_. The constitutive expression plasmid pPG-T7g10-PPT (Figure 1Aa), was constructed in our laboratory.

### 2.2. Construction of Recombinant Lactic Acid Bacteria

A schematic diagram for the construction of recombinant plasmids is shown in Figure 1A. In brief, following genomic RNA extraction from PEDV propagated in Vero cells, the gene encoding the COE antigen of PEDV was obtained by reverse transcription (RT)-PCR, and the gene encoding DCpep was then fused to 3′ terminus of the *COE* gene by fusion PCR. DC-targeting peptides (DCpep) that specifically bound to human DCs after screening a 12-mer peptide phage display library [7]. In this study, primer pairs F1/P1 and F1/DCpep (Table 1) were used to amplify *COE* gene and *COE-DCpep*, respectively.The gene *COE* and the fusion gene *COE-DCpep* were then cloned into the expression plasmid pPG-T7g10-PPT, giving rise to recombinant plasmids pPG-T7g10-PPT-COE (Figure 1Ab) and pPG-T7g10-PPT-COE-DCpep (Figure 1Ac), respectively. Details of the primers used in this study are listed in Table 1. The recombinant plasmids pPG-T7g10-PPT-COE and pPG-T7g10-PPT-COE-DCpep were then transformed into *L. casei* 393 competent cells by electroporation [22], to generate recombinant strains pPG-COE/L393 and pPG-COE-DCpep/L393, respectively.

### 2.3. Protein Expression

The bacterial strains pPG-COE-DCpep/L393, pPG-COE/L393, pPG/L393 and *L. casei* 393 were cultured overnight in MRS broth and harvested by centrifugation at 9000× *g* for 10 min at 4 °C. After cell lysis and centrifugation, the supernatant was subjected to 12% sodium dodecyl sulfate-polyacrylamide gel electrophoresis (SDS-PAGE) and western blot assay. The proteins in the supernatant were separated by SDS-PAGE, electrotransferred onto PVDF membranes (Millipore, Milford, MA, USA), and the immunoblot was then developed using a mouse anti-COE monoclonal antibody (dilution at 1:500) prepared in our laboratory as the primary antibody, and horseradish peroxidase (HRP)-conjugated goat anti-mouse IgG antibody (dilution at 1:3000) (Sigma, Ronkonkoma, NY, USA) as the secondary antibody. The results were then visualized using a chemiluminescent substrate reagent (Thermo Scientific, Durham, NC, USA) according to the manufacturer’s instructions. 

Moreover, in order to further analyze whether the protein of interest was displayed on the cell surface of the recombinant strains pPG-COE-DCpep/L393 and pPG-COE/L393, an immunofluorescence assay was performed according to a previously described method [24]. Briefly, the recombinant strains pPG-COE-DCpep/L393 and pPG-COE/L393 were cultured overnight in MRS at 37 °C. After centrifugation at 12,000× *g* for 5 min, the cell pellets were incubated with mouse anti-COE monoclonal antibody (dilution at 1:100) and fluorescein isothiocyanate (FITC)-labeled goat anti-mouse IgG (Invitrogen, Carlsbad, CA, USA) (diluted at 1:4000). After washing three times with phosphate buffered saline (PBS), the nuclei were counterstained with 4′,6′-diamidino-2-phenylindole (DAPI) (Invitrogen, Carlsbad, CA, USA) for 30 min at 4 °C. The pellets were washed thrice, resuspended in 1 mL PBS and smeared on a microscope slide. Fluorescence images were acquired by laser confocal microscopy (Zeiss, Oberkochen, Germany).

### 2.4. Immunization and Specimen Collection

All the experimental procedures and animal management procedures were approved by the Institutional Committee of the Northeast Agricultural University for the Animal Experiments (2016NEFU-315, 13 April 2017), Harbin, China. Six-week-old female specific pathogen-free (SPF) BALB/c mice (*n* = 60) were obtained from Liaoning Changsheng Biotechnology Co., Ltd. (Benxi, China) and kept under SPF conditions for one week with free access to a standard chow diet and water in accordance with institutional guidelines.Prior to oral administration, the recombinant strains weregrown in MRS broth at 37 °C for 12 h, and the cells were then washed twice with PBS, and resuspended in PBS to a final concentration of 10^10^ CFU/mL. The mice were orally dosed with 100 μL of pPG-COE-DCpep/L393 and pPG-COE/L393groups (15 mice per group). The control groups of mice (15 mice per group) were orally administered with an equivalent dose of pPG/L393 and PBS. The immunization protocol was performed on three consecutive days (days 1, 2, and 3), and a booster immunization was given on days 15, 16, and 17 (Figure 1B). Specimen collection was then performed at different time points post immunization (Figure 1B). Serum samples were collected from immunized mice on days 0, 10, 17, 27, 34, and 41 after immunization and stored at −20 °C until used. Genital tract secretions were collected on days 0, 10, 17, 27, 34, and 41 after primary immunization and stored at −40 °C until analyzed by ELISA for SIgA levels. Fecal samples were used for detecting SIgA antibody were collected and treated according to methods described previously with slight modifications [25]. In brief, 0.1 g of fecal pellets were sampled and subsequently suspended in 400 μL of PBS containing 1 mmol/L phenylmethylsulfonyl fluoride (Sigma, Ronkonkoma, NY, USA) and 1% bovine serum albumin (BSA). Then, after incubating at 4 °C for 16 h and centrifugation at 12,000× *g* for 5 min, the supernatants were stored at −20 °C until used. In addition, on days 0 and 40, the intestinal mucus samples was gently scraped from the excised intestinal tissue with HEPES buffer, and clarified by centrifugation as described previously [26].

### 2.5. Activation of DC Costimulatory Molecules

To evaluate the potential effect of pPG-COE-DCpep/L393 on DCs, murine mesenteric lymph nodes (MLN) cells were collected at 36 h after orally inoculated with pPG-COE/L393, pPG-COE-DCpep/L393 according to methods described previously [27]. Briefly, MLN cells were prepared using a 70 mM pore filter (BD Falcon, Franklin Lakes, NJ, USA) by gentle crushing of organs. Single MLN cell suspensions were incubated with CD16/CD32 (BD PharMingen, San Diego, CA, USA) to block Fc receptors (FcR). After cell suspensions were washed and resuspended in complete RPMI 1640, the cells were analyzed for DC activation by staining with fluorescein isothiocyanate (FITC)-conjugated surface molecule CD11c, CD80, CD86 and MHC II (BD Biosciences, San Diego, CA, USA). Samples were examined with a FACSCalibur cytometer.

### 2.6. ELISA

The levels of anti-PEDV specific IgG antibody in the sera and SIgA antibody in the feces, genital tract secretions, and intestinal mucus samples collected from immunized mice at the indicated time points were determined by ELISA. Briefly, a polystyrene microtiter plate was coated overnight at 4 °C with PEDV propagated in Vero cells. After washing thrice with PBS-0.1% Tween 20, the plate was blocked with 5% skimmed milk at 37 °C for 2 h. After washing, the collected immune samples were added into the plate in triplicate and incubated at 37 °C for 1 h. After washing, HRP-conjugated goat anti-mouse IgG/IgA-specific antibody (Invitrogen, Carlsbad, CA, USA) was added to the plate and incubated at 37 °C for 1 h. Furthermore, the expression of DCpep was verified by ELISA with COE-DCpep antiserum, obtained from mice immunized with pPG-COE-DCpep/L393. Briefly, a polystyrene microtiter plate was coated with synthetic DCpep protein. After being washed and blocked, the two-fold serially dilutions of COE-DCpep antiserum were added into the plate in triplicate, and then HRP-conjugated goat anti-mouse IgG-specific antibody (Invitrogen, Carlsbad, CA, USA) was added to the plate.The substrate o-phenylenediaminedihydrochloride (Sigma, Ronkonkoma, NY, USA) was used for color development followed by determination of absorbance at 490 nm.

### 2.7. Determination of PEDV Neutralizing Activity of Antibodies

For determining the PEDV neutralizing activity of the serum antibodies obtained from the immunized mice, 50 μL of sera samples serially diluted two-fold, were each mixed with 50 μL of 200 50% tissue culture infective dose (TCID_50_) PEDV propagated in Vero cells and incubated at 37 °C for 1 h; the mixture was then transferred to Vero cell monolayers in 96-wellplates. After incubation at 37 °C in a 5% CO_2_ atmosphere for 3 days, PEDV-specific cytopathic effects (CPE) were observed. In this work, three technical replicates and eight biological replicates per sample were performed, and the positive serum control, negative serum control, virus control, and blank control were designed in parallel.

### 2.8. Lymphocyte Proliferation and Cytokine Detection

On day 40 after primary immunization, five mice from each group were sacrificed for preparing splenocytes under aseptic conditions to perform the lymphocyte proliferation assay, as previously described [28]. In brief, the splenocytes in quadruplicates at a concentration of 5 × 10^6^ cells/mL were cultured in RPMI-1640 plus 20% fetal calf serum at 37 °C with 5% CO_2_ in a 96-well plate. The cells were stimulated with 0.5 μg/mL and 5 μg/mL of purified PEDV COE protein (specific antigen stimulation) for 60 h at 37 °C. In parallel, stimulation with 5 μg/mL of concanavalin A (ConA) and culture medium was used as the positive control and negative control, respectively. With absorbance measured at 490 nm, lymphocyte proliferation was evaluated using the CellTiter 96 AQueous Non-Radioactive Cell Proliferation Assay (Promega, Madison, WI, USA)according to the manufacturer’s instruction.The stimulation index was calculated as follows: the mean reading of triplicate antigen stimulation wells divided by the mean reading of triplicate negative control wells.

For detecting the interferon-gamma (IFN-γ) and interleukin-4 (IL-4) levels in the culture supernatant of splenocytes, the culture supernatants were collected after antigen stimulation for 60 h, and used in OptEIA Set Mouse IFN-γ and IL-4 (Biosource InternationalInc., Camarillo, CA, USA) antigen-capture ELISA according to the manufacturer’s instructions. All assays were performed in quadruplicate. In the culture supernatants, the concentration of IFN-γ and IL-4was calculated using a linear-regression equation and the absorbance values of the standards [29].

### 2.9. Statistical Analysis

Data are shown as the means ± standard errors of three replicates per test in a single experiment repeated thrice. GraphPad Prism V5.0 (San Diego, CA, USA) was used to perform statistical analyses. Tukey’s multiple comparison tests and one-way analysis of variance (ANOVA) were used to analyze the significance of the difference between means. *p*-Values less than 0.05 (*p* < 0.05) were considered significant and *p*-values less than 0.01 (*p* < 0.01) as highly significant.

## 3. Results

### 3.1. Identification of the Protein of Interest Expressed by Recombinant L. casei

**Figure 2:**
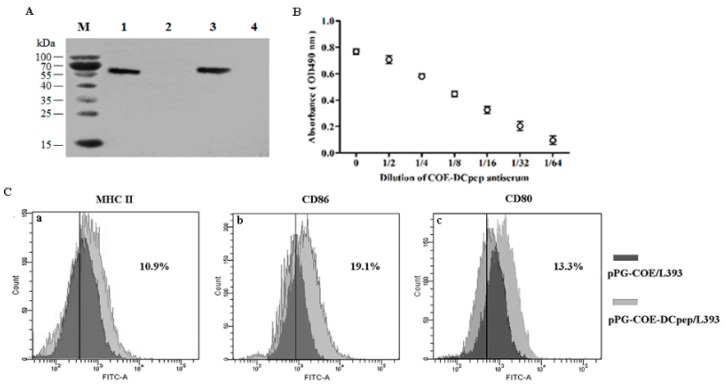
Expression of the protein of interest identified by western blot and ELISA (**A**,**B**), and activation of DC costimulatory molecules by *L. casei* 393 expressing pPG-COE-DCpep (**C**). The proteins in the lysate of overnight cultured *L. casei* 393, pPG/L393, pPG-COE/L393, and pPG-COE-DCpep/L393 were separated by sodium dodecyl sulfate-polyacrylamide gel electrophoresis (SDS-PAGE) followed by identification by western blot. Immunoblot with the expected sizes were respectively observed for recombinant strain pPG-COE/L393 (lane 1) and pPG-COE-DCpep/L393 (lane 3), but not for pPG/L393 (lane 2) and *L. casei* 393 (lane 4) (Figure 2A). M: protein molecular weight maker. Expression of DCpep protein were analyzed by ELISA (Figure 2B) in miceimmunizedthe recombinant *L. casei*, using synthetic DCpep protein as the coating antigen. DCs were isolated from the mesenteric lymph nodes (MLNs) of mice immunized with pPG-COE/L393, pPG-COE-DCpep/L393 at 36 h, and the expression levels of histocompatibility complex (MHC) II (Figure 2Ca), CD86 (Figure 2Cb) and CD80 (Figure 2Cc) were determinedby flow cytometry.

**Figure 3:**
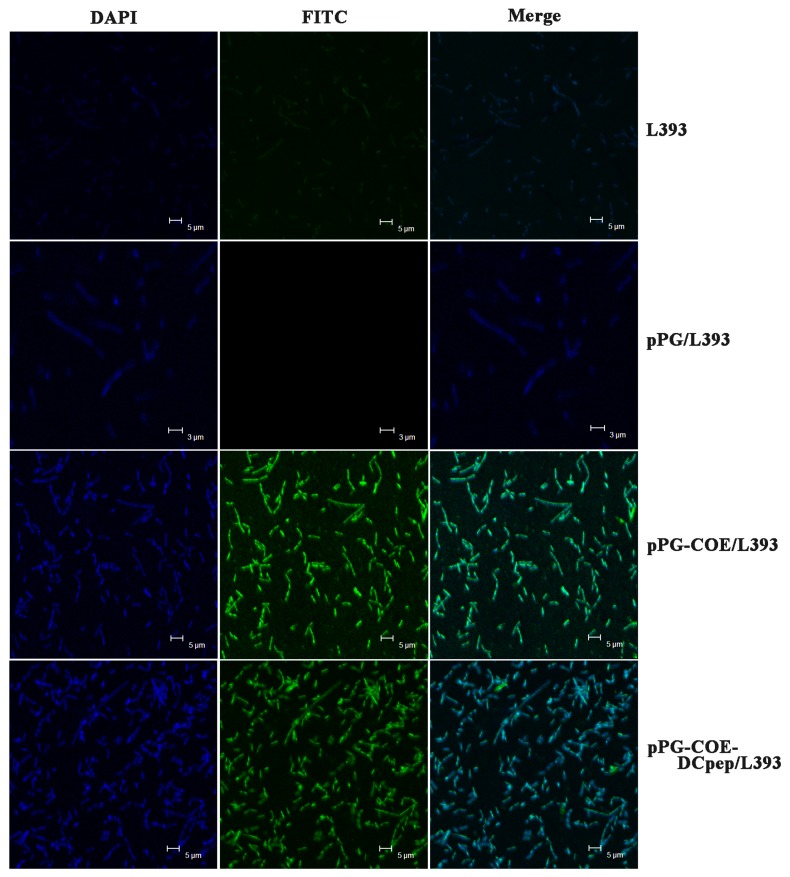
Cell surface display of protein of interest analyzed by immunofluorescence assay. Green fluorescence was only observed on the cell surface of pPG-COE/L393 and pPG-COE-DCpep/L393, and not on pPG/L393 and *L. casei* 393, indicating that the protein of interest was expressed and displayed on the cell surface of pPG-COE/L393 and pPG-COE-DCpep/L393.

Overnight cultures of pPG-COE-DCpep/L393, pPG-COE/L393, pPG/L393, and *L. casei* 393 were centrifuged, lysed, and analyzed by western blot assay. As shown in Figure 2A, a specific immunoreactive band of the expected size was observed in the lysate of the recombinant strain pPG-COE/L393 (lane 1) and pPG-COE-DCpep/L393 (lane 3), respectively, but not in pPG/L393 (lane 2) and *L. casei* 393 (lane 4), indicating that the protein of interest was efficiently expressed by the genetically engineered *L. casei* 393 strains. Moreover, an immunofluorescence assay was used to analyze the cell surface display of the protein of interest. Cells of overnight cultured *L. casei* 393, pPG/L393, pPG-COE/L393, and pPG-COE-DCpep/L393 were incubated with mouse anti-COE monoclonal antibody and FITC-conjugated goat anti-mouse IgG antibody, and then observed by laser confocal microscopy. As shown in Figure 3, green fluorescence observed on the cell surface of pPG-COE/L393 and pPG-COE-DCpep/L393, but not on pPG/L393 and *L. casei* 393. The results indicated that the protein of interest was expressed and displayed on the cell surface of pPG-COE/L393 andpPG-COE-DCpep/L393.

As shown in Figure 2B, DCpep protein can be detected with COE-DCpep antiserum which wasdiluted at 1:2–1:64. With the increase of dilution of COE-DCpep antiserum, the immunoreactive is weakened. Moreover, we showed that there is no cross-reactivity between the mouse anti-COE monoclonal antibody and synthetic DCpep protein.

### 3.2. Activation of DC Costimulatory Molecules by pPG-COE-DCpep/L393

To evaluate the potential effect of pPG-COE-DCpep/L393 on DCs, mice were orally immunized with pPG-COE/L393 and pPG-COE-DCpep/L393. The costimulatory molecules of DC were analyzed by staining with fluorescein isothiocyanate (FITC)-conjugated CD80, CD86 and histocompatibility complex (MHC) II, as shown in Figure 2C. The results showed that compared to the pPG-COE/L393, pPG-COE-DCpep/L393 induced a significantly increased in the surface expression of CD80^+^, CD86^+^ and MHCII^+^ on CD11c^+^ cells at 36 h post-vaccination in mice (the ratio (%) of the DC marker CD11c^+^ was >80%) which indicated that the oral vaccination of mice with pPG-COE-DCpep/L393 effectively elicited the activation of DCs in the MLNs.

### 3.3. IgG Levels Induced by Recombinant L. casei 393 via Oral Immunization

**Figure 4:**
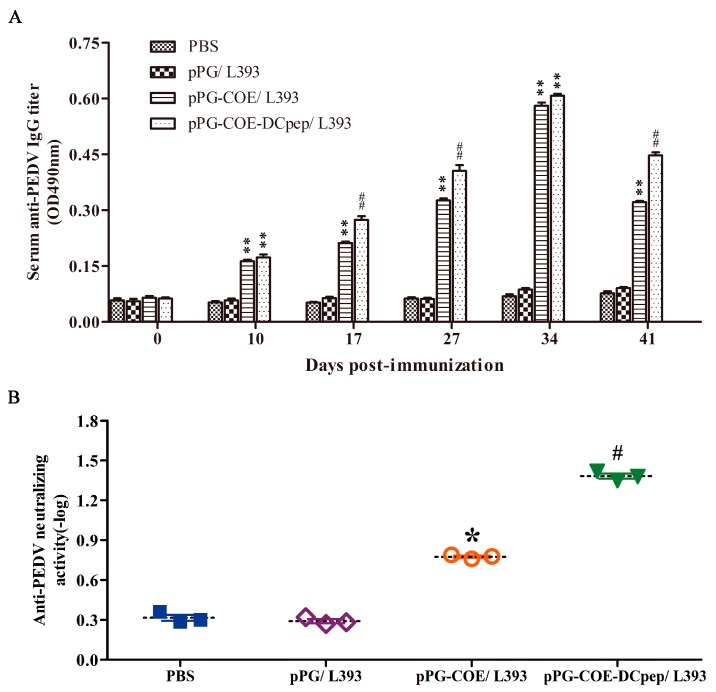
Determination of anti-porcine epidemic diarrhea virus (PEDV) specific IgG antibody and anti-PEDV neutralizing activity in mice post-immunization. Measurement of anti-PEDV IgG antibody (**A**) and anti-PEDV neutralizing activity (**B**) in sera from immunized mice by ELISA using PEDV as the coating antigen. Bars represent the mean ± standard error value of each group (* *p* < 0.05, ${}_{*}^{*}$ *p* < 0.01 compared to the control groups: pPG/L393 and PBS; ^#^ *p* < 0.05, ${}_{{}^{\#}}^{{}^{\#}}$ *p* < 0.01 compared to group: pPG-COE/L393).

The level of anti-PEDV IgG antibodies in mice induced by pPG-COE/L393 and pPG-COE-DCpep/L393 was determined by ELISA. As shown in Figure 4A, from the tenth day after primary vaccination, a significant level of anti-PEDV specific IgG antibody was induced in mice that were orally administered with pPG-COE/L393 and pPG-COE-DCpep/L393 (*p* < 0.01) compared to the pPG/L393 and PBS groups. Moreover, after the booster immunization, a higher level of anti-PEDV IgG antibody was elicited in mice orally immunized with pPG-COE-DCpep/L393 (*p* < 0.01) compared to the pPG-COE/L393 group, which indicated that the DC-targeting peptide used as a vaccine adjuvant could enhance the immunogenicity of the antigen and promote the vaccine immune efficiency. However, there was no significant difference in the IgG antibody level of the control groups before and after immunization.

Moreover, the anti-PEDV neutralizing activity of the serum antibody obtained from immunized mice was determined (Figure 4B). Results showed that the antibody response in mice that received pPG-COE-DCpep/L393 possessed a stronger anti-PEDV neutralizing activity (1:24) than that in mice orally administered with pPG-COE/L393 (1:6), pPG/L393 (1:2) and PBS (1:2). As might be expected, the *L. casei* oral vaccine (pPG-COE-DCpep/L393) expressing the DC-targeting peptide fused with PEDV COE antigen could effectively elicit an anti-PEDV immune response in vivo.

### 3.4. SIgA Levels Induced by Recombinant L. casei 393 via Oral Immunization

**Figure 5:**
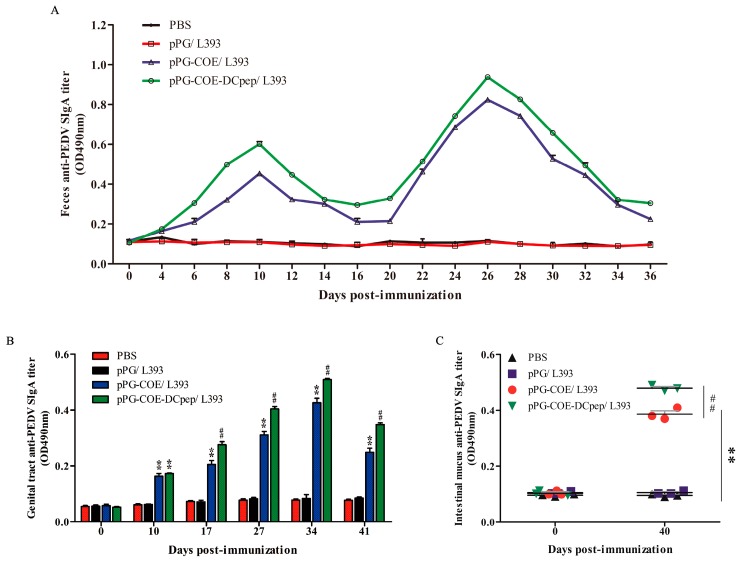
Determination of anti-PEDV mucosal SIgA antibody in feces (**A**), genital tract (**B**) and intestinal mucus (**C**) by ELISA using PEDV as the coating antigen. Bars represent the mean ± standard error value of each group (* *p* < 0.05, ${}_{*}^{*}$ *p* < 0.01 compared to the control groups pPG/L393 and PBS; ^#^ *p* < 0.05, ${}_{{}^{\#}}^{{}^{\#}}$ *p* < 0.01 compared to group pPG-COE/L393 in Figure 5B,C).

The intestinal mucosa cell-mediated immune response was further evaluated by measuring the specific anti-PEDV SIgA antibody in the feces, genital tract, and intestinal mucus collected from immunized mice post-immunization via ELISA. The mucosal SIgA levels increased after oral immunization with pPG-COE/L393 and pPG-COE-DCpep/L393 in the feces (Figure 5A), genital tract (Figure 5B), and the intestinal mucus (Figure 5C) (*p* < 0.01) compared to those in the control groups of mice orally immunized with pPG/L393 and PBS. Anti-PEDV SIgA in the feces could be detected as early as the sixth day post-immunization with pPG-COE-DCpep/L393 and the eighth day after primary vaccination with pPG-COE/L393, respectively; following a booster immunization, the SIgA levels significantly increased. There was no significant difference in the SIgA level observed in the control groups of mice before and after immunization (*p* > 0.05). In contrast, the level of SIgA antibody induced by pPG-COE-DCpep/L393 via oral immunization was significantly higher than that induced by pPG-COE/L393.

### 3.5. Lymphocyte Proliferation

**Figure 6:**
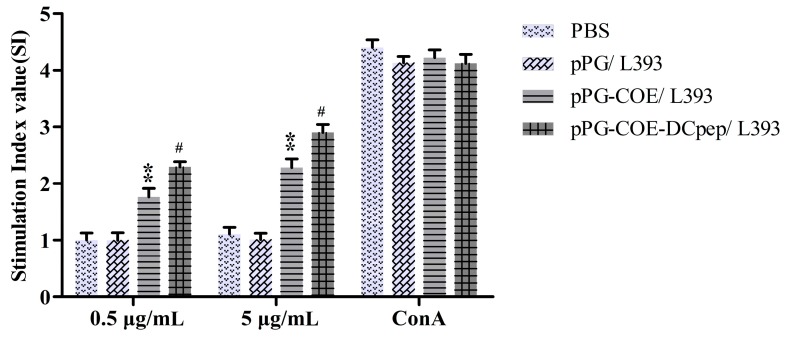
Lymphocyte proliferation in immunized mice determined by 3-(4,5-dimethylthiazol-2-yl)-2,5-diphenyltetrazolium bromide (MTT) assay in response to PEDV COE protein as a stimulating agent. Bars represent the mean ± standard error value of each group (* *p* < 0.05, ${}_{*}^{*}$ *p* < 0.01 compared to the control groups: pPG/L393 and PBS; ^#^ *p* < 0.05, ${}_{{}^{\#}}^{{}^{\#}}$ *p* < 0.01 compared to group: pPG-COE/L393).

Splenocytes were isolated from immunized mice and restimulated with the PEDV COE protein in vitro to test the lymphocyte proliferation response by 3-(4,5-dimethylthiazol-2-yl)-2,5-diphenyltetrazolium bromide (MTT) assay using Con A as the positive control and cell culture medium as negative control. As shown in Figure 6, upon stimulation with the purified PEDV COE protein, the stimulation index markedly increased in the group immunized with pPG-COE-DCpep/L393 compared to that in the groups immunized with pPG-COE/L393 (*p* < 0.05), pPG/L393 (*p* < 0.01) and PBS (*p* < 0.01), and showed a dose-dependent response.

### 3.6. Cytokine Levels

**Figure 7:**
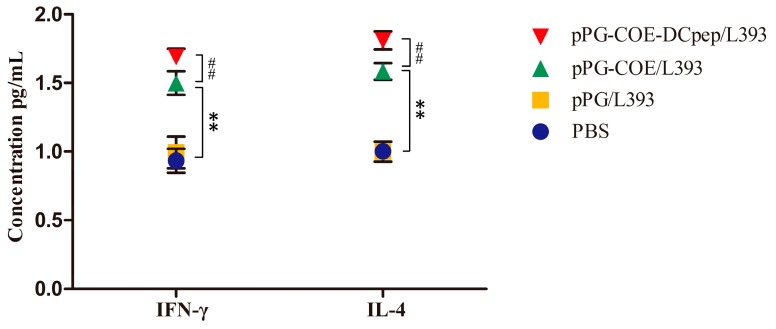
Determination of cytokines produced by splenocytes of mice orally administered with pPG-COE-DCpep/L393, pPG-COE/L393, pPG/L393, and PBS, respectively. Bars represent the mean ± standard error value of each group (* *p* < 0.05, ${}_{*}^{*}$ *p* < 0.01 compared to the control groups pPG/L393 and PBS; ^#^ *p* < 0.05, ${}_{{}^{\#}}^{{}^{\#}}$ *p* < 0.01 compared to group pPG-COE/L393).

As shown in Figure 7, in response to the PEDV COE antigen, splenocytes obtained from mice orally administered with pPG-COE-DCpep/L393 produced significant higher levels of the Th1-associated cytokine IFN-γ and the Th2-associated cytokine IL-4 than those in mice receiving pPG-COE/L393 (*p* < 0.01).

## 4. Discussion

Currently, porcine epidemic diarrhea caused by PEDV has become the one of the important swine diseases, resulting in huge economic loses for the pig industry. Since 2013, porcine epidemic diarrhea has spread throughout the United States and has greatly damaged the American pork industry [30]. The vaccination failure and high death rate seen in the 2010 Chinese outbreak with the re-emergence of a more highly virulent PEDV may have been due to changes in the antigenicity of PEDV based on amino acid mutations [31]. The emergence and re-emergence of PEDV suggests that it is able to elude current vaccine strategies [32]. However, the neutralizing antigenic epitopes of S protein of PEDV have been showed high cross-protection against variant strains [33]. Therefore, the use of core neutralizing epitope (COE) of PEDV isolates S protein to develop PED vaccines should be a useful approach for control of PED epidemics in the region. In this study, a PEDV isolate LJB/15 was used and the nucleotide sequence encoding the *COE* of PEDV LJB/15 showed more than 98% identity with other PEDV strains isolated in China in recent years.

The mucosal immune system, based mostly on SIgA, is the first and a major barrier against invading pathogens targeting the gastrointestinal tract [23]. Therefore, it is necessary to develop oral mucosal vaccines that can induce strong and protective immune responses in the mucosa (to prevent initial infection and pathogen replication), predominantly by means of SigA [34]. *L. casei* 393, as the antigen delivery carrier used in our study, can adhere to and colonize intestinal mucosa of murine and pigs and to tolerate bile, thus serving as a promising candidate for the delivery of antigenic material to the mucosa in some vaccines [35,36]. In order to enhance the delivery efficiency of the antigen of interest and reduce the number of vaccinations and the quantity of lactic acid bacteria required for oral administration, intestinal DC-targeting mucosal vaccines were suggested as a promising approach of oral vaccination to induce high mucosal immune responses [10].

In this study, a genetically engineered *Lactobacillus casei* oral vaccine (pPG-COE-DCpep/L393) expressing a DC-targeting peptide fused with PEDV COE antigen was developed. Expression of the fusion protein was identified by western blot and immunofluorescence assays. The immunogenicity of the orally administered pPG-COE-DCpep/L393 was evaluated in mice, and the results showed that oral immunization with pPG-COE-DCpep/L393 can induce a significant level of anti-PEDV systemic IgG and mucosal SIgA antibody responses, indicating a potential vaccine strategy against PEDV infection.

The antigens of interest intracellularly biosynthesized by lactic acid bacteria could not effectively induce antigen-specific immune responses via oral administration, due mainly to the target antigens cannot effectively interact with intestinal mucosa tissues, in particular DC cells [37]. Thus, in this study, we used the pPG-T7g10-PPT expression vector that was a constitutive cell surface expression plasmid developed by our lab to construct recombinant lactic acid bacteria for the delivery of vaccine antigen. Remarkably, pPG-T7g10-PPT exhibiting a significant advantage as compared with inducible gene expression systems, which require the use of an inductive agent. Additionally, we used phosphatidylglycerol phosphate synthase (pgsA) from *Bacillus subtilis* subsp. *chungkookjang* as an anchor protein and fused the COE protein to its C terminus within pPG-T7g10-PPT expression vector so that it can anchor the protein to the bacterial surface without a sortase [38], thereby enhancing the immunogenicity. Therefore, in this study, the constitutive cell surface expression system serves as a powerful tool for the construction of recombinant *Lactobacillus* oral live vaccines.

It is now clear that the intestinal mucosa is the primary site of PEDV infection and transmission. Thus, an effective mucosal immune response requires secretion of both serum IgG and mucosal SIgA. Our data showed that a strong and long-lasting anti-PEDV IgG response was detected in mice orally immunized with pPG-COE-DCpep/L393 and that the anti-PEDV IgG response increased rapidly following a booster immunization. Although the SIgA antibody represents the major humoral mechanism of defense of mucosal surfaces, serum-derived IgG can also contribute significantly to immune defense, by reducing the ability of pathogens to cross the intestinal mucosa and the systemic spread of invasive pathogens, and thereby supplements the mucosal protection provided by SigA [7,39]. Compared with systemic immunity, the distinctive hallmark of mucosal immunity is the local production of SIgA, which can prevent binding of pathogens to the intestinal epithelial cells and remove pathogens crossing the gastric mucosal barrier [40]. Significantly, the critical advantage of the lactic acid bacteria vaccine is the ability to induce antigen-specific IgA responses in the intestinal mucosa. In this study, we observed here that oral administration of strain pPG-COE-DCpep/L393 expressing a DC-targeting peptide fused with PEDV COE antigen induced substantially elevated levels of anti-PEDV specific SIgA response in the feces, genital tract secretions, and intestinal mucus samples.Ourresults showed that oral administration of strains pPG-COE-DCpep/L393 induced a higher response than did strains pPG-COE/L393 expressing COE antigen only. However, despite statistical significance, the difference is not very large compared to the difference between a positive response and negative controls. On one hand, it may be relevant in mice species, so it need to further studies to show relevance in other species. On the other hand, in this study, the efficient antigen-specific immune responses of PEDV only induced by COE antigen, the presence of DCpep further potentiated the effect of recombinant COE antigen.

Extracellular cytokine analysis is a key method for evaluating the ability of a vaccine to elicit Th1 or Th2 responses [23]. Cytokines produced by CD4^+^ T helper (Th) lymphocytes could regulate immune system functions, including antibody production and cellular immune responses. The Thl cell response is closely related to cellular immune responses, whereas the Th2 cell response is associated with humoral immunity. The IL-4/IFN-γ ratio is used to indicate the Th1 or Th2 bias of the generated immune response [41]. In this study, we determined the levels of IFN-γ and IL-4 in the immunized mice, and observed that oral immunization with pPG-COE-DCpep/L393 can significantly elicit IFN-γ and IL-4 production in a ratio of about 1:1, which indicates that pPG-COE-DCpep/L393 can induce the development of both Thl- and Th2-type immune responses in vivo, similar to the previous finding that use of the DCpep ligand supported both Thl- and Th2-type immune responses [23]. Recently, induction of SIgA against microbes and foreign immunogens was demonstrated to be a T cell-dependent immune response [42]. In this work, the fusion protein COE-DCpep delivered by *L. casei* canactivate DC, then elicit both Thl- and Th2-type immune responses, indicating that efficient T cell-mediated immunity was critical for SIgA induction. Moreover, pPG-COE-DCpep/L393 can also elicit a more efficient and robust lymphocyte proliferation response against PEDV COE antigen.

The translational value of studies in mice to the pig species is significant. Many vaccines regarding piglet diarrhea such as PEDV and TGEV were developed in the mice model [21,43,44,45]. Jiang et al. showed that oral *L.casei* vaccine against TGEV in mice could increase the Treg population [22]. To further evaluate the immune effect of the vaccine in the target animal, Jiang et al, using piglets as models, suggested that the oral *L. casei* vaccine elicit effective mucosal and systemic immune responses against TGEV infection [46]. We believe that this translational value of the oral *L. casei* vaccine against TGEV from mice to pig has already provided promising results, especially for the congeneric PEDV which also causes pig diarrhea. Furthermore, DCpep recognizes human, avian, canine, equine, feline and mouse’s DCs [10]. So we hypothesize DCpep also bound to pig DCs due to the similarity of the immune system between pig and human. Although mice are not susceptible to PEDV infection, good immune responses of the *L.casei* vaccine in mice were observed in this study. The results of our study at least demonstrate that *L. casei* surface-displayed PEDV core neutralizing epitope (COE) fused with DC-targeting peptide (DCpep) could act as a novel mucosal vaccine formulation and provide a useful strategy to induce efficient immune responses against PEDV infection. Certainly, further investigations are indispensable to evaluate the immunogenicity and host immunity protection of this vaccine following its oral administration in piglets.

In conclusion, an intestinal DC-targeting oral vaccine strategy using *Lactobacillus casei* to deliver the core neutralizing epitope (COE) antigen of PEDV conjugated with a DC-targeting peptide as an immune adjuvant was explored to develop an anti-PEDV vaccine for oral administration in this study. We demonstrated that the genetically engineered pPG-COE-DCpep/L393 can efficiently induce mucosal, humoral, and cellular immune responses against PEDV, suggesting a promising vaccine strategy.

## Figures and Tables

**Table 1:** Details of primers used in this study.

Target	ID	Primer Sequence (5′–3′)	PCR Size	Accession No./Reference
COE	F1	GAGCTC^a^**GATTATAAGGATGACGATGACAAG**^b^AAGCTTGTTACTTTGCCATCGTTT	465 bp	JX512907
	P1	GGGCCC^a^TCAAACGTCCGTGACACCTTC		
COE-DCpep	F1	GAGCTC^a^**GATTATAAGGATGACGATGACAAG**^b^AAGCTTGTTACTTTGCCATCGTTT	507 bp	Shi et al., 2016 [23]
	DCpep	GGGCCC^a^TTA*TGGACGTTGTGGTGTTGAATGATATGATGGATAAAA*^c^TCTAGA^a^		

^a^ Restriction enzyme recognition sites used for cloning; ^b^ Flag tag are shown in bold; ^c^ DCpep sequences are shown in italic.
